# Molecular and Biochemical Evidences for Beneficial Effects of Zinc Oxide Nanoparticles in Modulation of Chlorpyrifos Toxicity in Human Lymphocytes

**Published:** 2018

**Authors:** Mona Navaei-Nigjeh, Mahdi Gholami, Maryam Sadat Fakhri-Bafghi, Maryam Baeeri, Mohammad Abdollahi

**Affiliations:** a *Department of Tissue Engineering and Applied Cell Sciences, School of Advanced Technologies in Medicine, Tehran University of Medical Sciences, Tehran, Iran.*; b *Toxicology and Diseases Group, The Institute of Pharmaceutical Sciences (TIPS), Tehran University of Medical Sciences, Tehran, Iran. *; c *Department of Toxicology and Pharmacology, Faculty of Pharmacy, Tehran University of Medical Sciences, Tehran, Iran. *; d *School of Medicine, Tehran University of Medical Sciences, Tehran, Iran.*

**Keywords:** Chlorpyrifos, Human lymphocytes, Organophosphorus pesticide, Oxidative stress, Zinc oxide nanoparticles

## Abstract

Chlorpyrifos (CP), an acetylcholinesterase (AChE) inhibitor, is used throughout the world as an insecticide in agriculture and an eradicating agent for termites around homes. In the present study, we examined the protective role of zinc oxide nanoparticles (ZnO NPs) in human CP-treated lymphocytes. Lymphocytes isolated by Ficoll and exposed to 75 µg/mL CP either alone or in combination with logarithmic doses of ZnO NPs (0/1, 1, 10, 100 µg/mL). After a 3-day incubation period, the viability and oxidative stress markers were determined. Then, the levels of tumor necrosis factor-α (TNF-α), as an inflammatory index along with AChE activity and cell death were evaluated. Our results showed that incubation with CP significantly increases the percent of cell death, activities of caspase-3 and -9, level of TNF-α and also promotes the levels of biomarkers which play important role in oxidative stress. On the other hand, the activity of AChE and levels of the total antioxidant power (TAP) decreased in CP-treated lymphocytes. In contrast, lymphocytes treated with different concentrations of ZnO NPs showed a significant decrease in the percent of mortality as well as the levels of TNF-α, as compared with CP-treated lymphocytes. Besides, ZnO NPs increased the levels of AChE and TAP at 1 µg/mL. In conclusion, the results indicate the protective effects of ZnO NPs in the prevention of cytotoxic activity of CP in the lymphocytes.

## Introduction

Organophosphorus (OP) pesticides are used commonly as an agricultural pesticide nowadays. Chlorpyrifos [O,O-diethyl-O-(3,5,6-trichloro-2-pyridyl)-phosphorothioate] (CP) is an example of them, whose toxic effects have been proven in different tissues and organs (1). All OPs including CP can inhibit acetylcholinesterase (AChE) and interrupt both central and peripheral neurotransmitter- mediated effects (2). Induction of oxidative stress, disruption of mitochondrial ATP production in cells, generation of reactive oxygen species (ROS), reduction of antioxidant defense enzymes and glutathione (GSH), and increase in cellular lipid and protein oxidation are other effects that may be seen by CP (3-6). Previous *in-vivo* studies have shown that OP compounds can interfere with the immune system (7-9). In this respect, tumor necrosis factor-α (TNF-α) is one of the most important immunologic mediators secreted from lymphocytes and monocytes which initiate oxidative stress and cell apoptosis (10). Exposure of lymphocytes with OPs such as CP can promote this process (11, 12).

Concurrent with the development of nanotechnology, nanoparticles (NPs) have been used to revolutionize several fields of science, such as methods of medical diagnosis and drug delivery during the last years. Among all NPs, metal NPs are more versatile and ZnO NPs are the most functional example of them. Zinc is an essential trace element that plays an important role in regulating cellular metabolism, especially in the immune system (13). Moreover, zinc plays an essential biochemical function that retards the oxidative processes (14). Although changing the compound to nanoform is not always beneficial regarding toxicological issues, nanoforms are able to reach more to organelles within the body where their efficacy may be increased. In this regard, previous studies showed that ZnO NPs are systemically absorbed, which elevates the zinc level in different tissues. Nevertheless, there are some controversies about toxic or non-toxic effects of ZnO NPs according to dose, size, shape, and cells affected (15). In this respect, although most studies have shown that high doses of ZnO NPs increase cell death while at low doses (<10 µg/mL) it has no negative effect on the viability and function of cells. For example, Sharma *et al*. exposed human liver cells to different concentrations of ZnO NPs (0.8–20 μg/mL) for 6, 12, and 24 h and showed that only 14 and 20 μg/mL of ZnO NPs after 12 and 24 h, have significant cytotoxicity and genotoxicity (16). In other studies, it was indicated that ZnO NPs induced apoptosis in human alveolar adenocarcinoma (A549) and human skin melanoma (A375) cells through oxidative stress via mitochondrial- and caspase-dependent pathways, significantly at concentrations higher than 5 μg/mL (17, 18). Taken together, the results of previous studies demonstrate that metal oxide NPs such as ZnO induce a range of biological responses that vary from cytotoxic to cytoprotective dependent on the size, charge, solubility, concentration, and the time which can be optimally adjusted based on the special purpose of study (19, 20).

Given the above evidence, the present endeavor was undertaken to develop a cell model using lymphocytes to evaluate compatibility of ZnO NPs in low concentrations. It is also aimed to investigate the possible modulatory role of ZnO NPs against CP-induced oxidative stress and determine the mechanisms lying behind this protection by viability and biochemical assays. 

## Experimental

*Chemicals*

**Figure 1:**
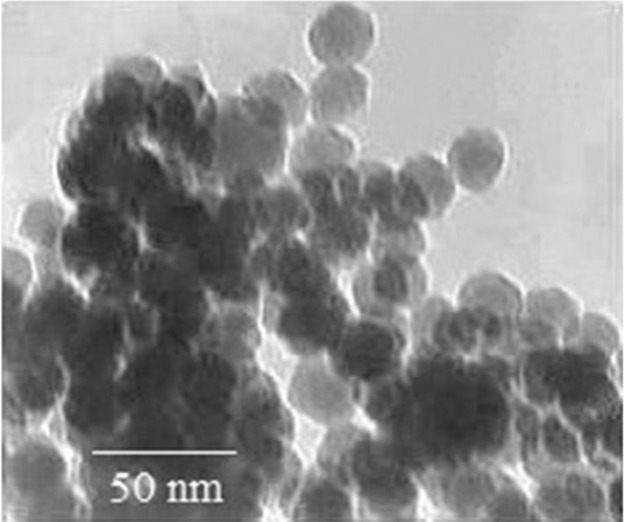
Transmission electron microscopy image of zinc oxide nanoparticles. The picture has been reproduced by permission from the Springer (License number 4082521300825) (47).

All chemicals were purchased from Sigma-Aldrich Chemie (Germany) unless human specific TNF-α enzyme-linked immunosorbent assay (ELISA) kit which was purchased from Bender MedSystems® (Austria) and ApoFlowEx® FITC Kit from Exbio (Czech Republic). ZnO NP (<100 nm) solution was purchased from Nano Zino (Tehran, Iran; http://nanozino.com) which its transmission electron microscopy image has been shown in the Figure 1 provided by the seller company.

*Human volunteer*

Venous blood samples were collected from twenty-five year old healthy volunteers without any history of smoking and consumption of medications. To prevent the process of coagulation, the samples were heparinized in a sterile situation. The study was approved by the Institute Review Board.

*Lymphocyte separation and culture*

Lymphocytes were separated from blood samples with the Ficoll-Paque procedure as described previously (11). Briefly, the blood samples were centrifuged at the experimental condition described previously and the lymphocytes were collected from the layer between plasma and Ficoll-Paque layers. Following separation, the cells were washed twice in phosphate buffer saline (PBS), and then cultured at 37 °C and 5% CO_2_ in Roswell Park Memorial Institute (RPMI) 1640 medium supplemented with 10% fetal bovine serum (FBS), 2 mM L-glutamine, and 100 µg/mL penicillin–streptomycin and followed by the addition of 50 μL/mL lipopolysaccharide (LPS) for cell growth stimulation. Viability, checked by analyzing the ability to exclude the dye trypan blue, always exceeded 90%.

*Treatment conditions & experimental groups*

According to a previous study (21), we used 75 μg/mL as the concentration of CP that induced oxidative stress in lymphocytes. In this regard, cell suspension (3×10^6^ cells/well) was incubated with culture medium in combination with 75 µg/mL CP for 72 h at 37 °C and 5% CO_2 _humidified atmosphere. For protective treatment, optimization of dose was done by treating CP-induced cells with logarithmic concentrations (0.1, 1, 10 and 100 μg/mL) of ZnO NPs for 72 h to ascertain the most effective dose. To fulfill this purpose, all the cells were split into half a dozen groups of four per each interval (n = 4). Treatment conditions of experimental groups are detailed in Table 1. After a 72-h period, the cell suspensions in all groups were centrifuged. The supernatant solutions were removed for the biochemical assays and the deposited cells were used for cellular assays in the succeeding measure.

**Figure 2:**
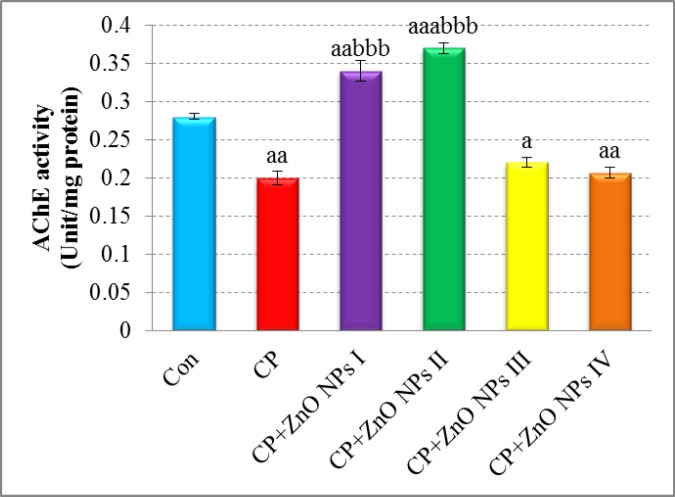
Effects of various concentrations of ZnO NPs in acetylcholinesterase (AChE) activity of isolated human lymphocytes in the presence of CP. Data are expressed as mean ± SEM. Significantly different from control at ^a^*p *< 0.05, ^aa^*p *<0.01, ^aaa^*p *< 0.001. Significantly different from CP at ^bbb^*p *< 0.001.

**Figure 3:**
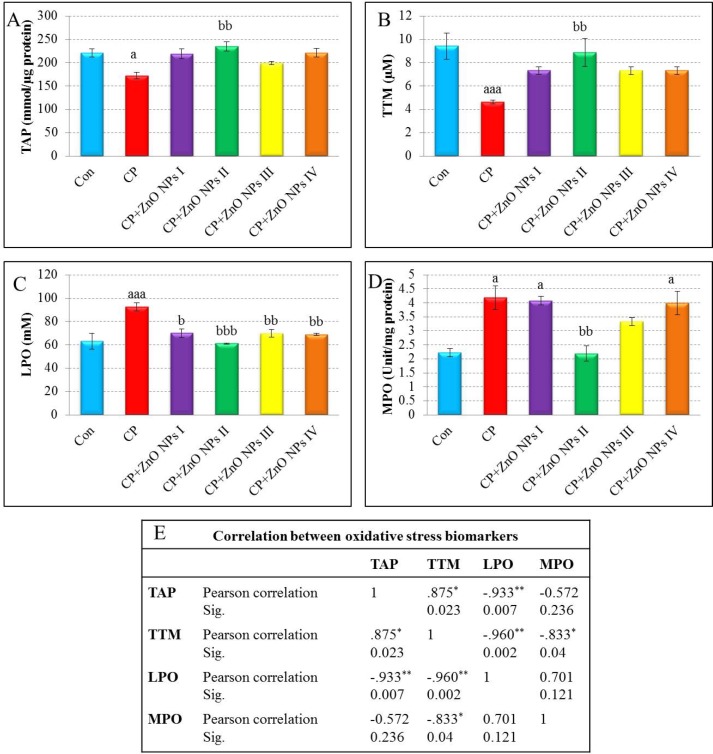
Effects of various concentrations of ZnO NPs in oxidative stress biomarkers [TAP values (A), TTM levels (B), LPO levels (C), and MPO activity (D)] of isolated human lymphocytes in the presence of CP. Data are expressed as mean±SEM. Significantly different from control at ^a^*p *< 0.05, ^aaa^*p*< 0.001. Significantly different from CP at ^b^*p*< 0.05, ^bb^*p *< 0.01, ^bbb^*p *< 0.001.

**Figure 4:**
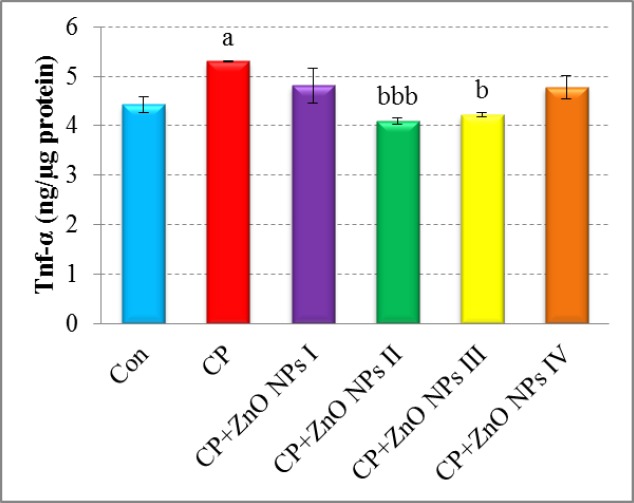
Effects of various concentrations of ZnO NPs in TNF-α release of isolated human lymphocytes in the presence of CP. Data are expressed as mean±SEM. Significantly different from control at ^a^*p *< 0.05. Significantly different from CP at ^b^*p* < 0.05, ^bbb ^*p* < 0.001.

**Figure 5:**
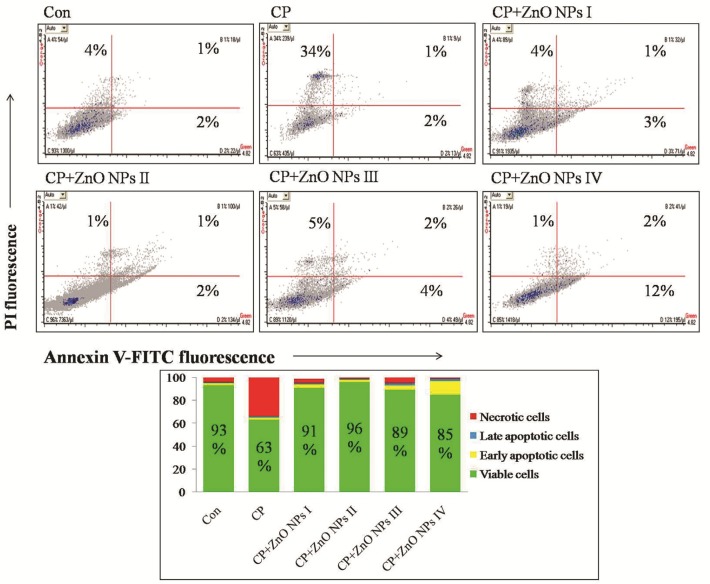
Flow cytometric analyses of apoptosis and necrosis in human lymphocytes induced by CP, alone or in combination with various concentrations of ZnO NPs using Annexin V-FITC and PI double staining.

**Figure 6:**
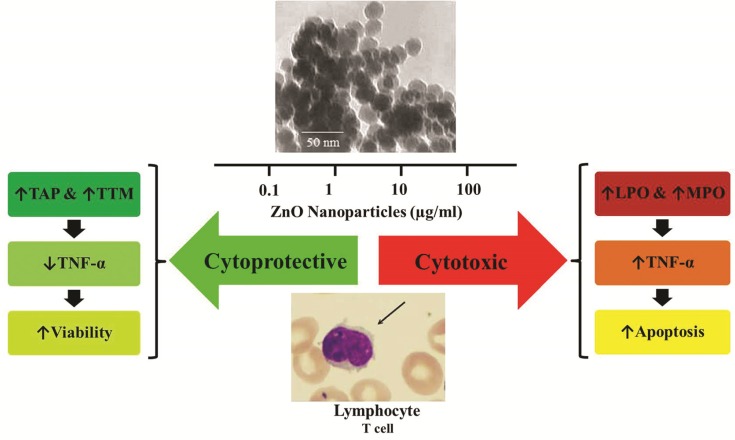
Summary of the study

**Table 1:** Treatment conditions of experimental groups

**Experimental groups**	**Treatment conditions**
Control	Lymphocytes in RPMI-1640 medium
CP	Lymphocytes in RPMI-1640 medium + CP (75 μg/mL)
CP+ZnO NPs I	Lymphocytes in RPMI-1640 medium + CP (75 μg/mL) + ZnO NPs (0.1 μg/mL)
CP+ZnO NPs II	Lymphocytes in RPMI-1640 medium + CP (75 μg/mL) + ZnO NPs (1 μg/mL)
CP+ZnO NPs III	Lymphocytes in RPMI-1640 medium + CP (75 μg/mL) + ZnO NPs (10 μg/mL)
CP+ZnO NPs IV	Lymphocytes in RPMI-1640 medium + CP (75 μg/mL) + ZnO NPs (100 μg/mL)

**Table 2:** Effects of various concentrations of ZnO NPs on mitochondrial, caspase-3 and -9 activities of isolated human lymphocytes in the presence of CP

	**Mean±SEM of**		
	**Mitochondrial activity** **(% of control)**	**Caspase-3** **activity** **(% of control)**	**Caspase-9** **activity** **(% of control)**
Control	100 ± 2.31	100 ± 1.41	100 ± 2.12
CP	63 ± 2.18^aa^	131 ± 1.39aaa	125 ± 2.81aaa
CP+ZnO NPs I	88 ± 7.45	113 ± 0.93^aa^,bbb	118 ± 5.62^aa^
CP+ZnO NPs II	125 ± 1^a^,bbb	108 ± 0.46bbb	108 ± 1^b^
CP+ZnO NPs III	88.5 ± 8.84	127 ± 2.32aaa	125 ± 0.77aaa
CP+ZnO NPs IV	86 ± 8.7.11	143 ± 2aaa,^b^	137 ± 3aaa

Significantly different from control at

Significantly different from CP at

a *p* < 0.05,

aa *p* < 0.01,

aaa *p* < 0.001.

b *p* < 0.05,

bbb *p *< 0.001.

*Determination of AChE activity*

AChE activity in lymphocytes was measured according to the modified Ellman method using acetylthiocholine iodide as the substrate and 5-5-bis dithionitrobenzoic acid (DTNB) as a coloring agent (22). Enzyme activity was expressed as Unit/mg protein.

*Oxidative stress assays*

To measure the oxidative stress of cells, total antioxidant power (TAP), total thiol molecules (TTM), lipid peroxidation (LPO), and myeloperoxidase (MPO) activity were determined.

*Determination of TAP*

The total antioxidant capacity of samples were determined by measurement of their abilities to reduce Fe^3+^ to Fe^2+^ by the ferric reducing antioxidant power (FRAP) test as previously set up and described (12). The FRAP assay measures the changes in absorbance at 593 nm owing to the formation of a blue colored Fe (II)-tripyridyltriazine compound from Fe (III) by the action of electron donating antioxidants. In this test, the results were expressed as mmol/μg protein.

*Determination of TTM*

To determine the level of TTM in the control and test groups, 5,5’-dithiobis-(2-nitrobenzoate) (DTNB) was used as a reagent. DTNB reacts with thiol molecules and creates a yellow complex, which has a good absorbance at 412 nm in a spectrophotometer as previously set up and described (9). The data were shown as µM.

*Determination of LPO*

For measuring the rate of LPO, thiobarbituric acid (TBA) was used which reacts with lipid peroxide molecules. The samples were mixed with trichloroacetic acid (TCA) (20%) and the precipitate was dispersed in H_2_SO_4_ (0.05 M). TBA (0.2% in sodium sulfate 2 M) was added and heated for 30 min in a boiling water bath. TBA-reactive substance adducts were extracted by n-butanol, and the absorbance was measured at 532 nm. This reaction forms in acidic pH and high temperature and the maximum absorption is a pink complex in 532 nm (23). The results were reported as mM.

*Determination of MPO activity*

As previously set up and described (24), 100 µL of preserved supernatant was mixed with 2.9 mL phosphate buffer containing 0.167 mg/mL O-dianisidine dihydrochloride and 0.0005% hydrogen peroxide. Then, MPO activity was measured spectrophotometrically as the change in absorbance at 460 nm. MPO activity was reported as Unit/mg protein.

*Determination of TNF-α*

Quantitative detection of TNF-α level in the supernatant of lymphocyte culture was performed using a human specific TNF-α ELISA kit and according to manufacturer manual. The absorbance of the final colored product was measured at 450 nm as the primary wavelength and 620 nm as the reference wavelength. TNF-α levels were expressed as ng/µg protein.

*Protein assay*

According to Bradford method, following binding to proteins, the maximum absorbance of the colored reagent Coomassie Brilliant Blue changes from 465 nm to 595 nm and the latter is measured spectrophotometrically (25). The standard curve was obtained from various concentrations of bovine serum albumin (BSA) as the standard. The data were expressed as 

mg/mL.

*Cellular assays*

*Determination of mitochondrial activity*

This assay is based on the reduction of3-4,5-dimethylthiazol-2-yl-2,5-diphenyltetrazolium bromide (MTT), a yellow tetrazole to purple formazan by mitochondrial respiration in viable cells. After 72 h incubation and centrifugation, the precipitated lymphocytes were washed twice with PBS. Then, 50 µL of MTT solution was added. After 4 h of incubation at 37 °C and 5% CO_2_ humidified atmosphere, colored crystals of formazan were dissolved with a 150 µL of dimethyl sulfoxide (DMSO) solution. The plates were kept on orbital shaker for 10 min and optical density (OD) was read by a multi-well scanning spectrophotometer (ELISA reader) at 570 nm as described previously (6). The viability of the treatment groups was expressed as the percentage of control 

which put on 100%.

*Caspase-3 and -9 activities assays*

Caspase-3 and -9 activities were measured by colorimetric assays based on the identity of specific amino acid sequences by these caspases as previously described (26). The tetrapeptide substrates were labeled with the chromophore r-nitroaniline (ρNA). ρNA is released from the substrate upon cleavage by caspase and produces a yellow color that is monitored by an ELISA reader at 405 nm. The amount of caspase activity present in the sample is proportional to the quantity of yellow color produced upon cleavage (27). In this test, the caspase-3 and -9 activities of the treatment groups were expressed as the percentage of controls which put on 100%.

*Determination of cell death (apoptosis vs. necrosis)*

To find out the mode of lymphocyte cell death (apoptosis or necrosis) induced by CP in the presence and absence of ZnO NPs, the Annexin V-FITC/propidium iodide (PI) staining was carried out using ApoFlowEx® FITC Kit obtained from Exbio (Czech Republic) according to manufacturer manual. The cells were scanned for fluorescence intensity in FL-1 (FITC) and FL-2 (PI) channels. The fraction of cell populations in different quadrants was analyzed using quadrant statistics. The values shown in the lower left, lower right, upper left, and upper right quadrants of each panel represent the percentage of viable, early apoptotic, necrotic, and late apoptotic (post-apoptotic necrotic) cells, respectively (28). Flow cytometric analysis was performed with flow cytometer (Mindray, China).

*Statistical analysis*

The results were presented as mean ± SEM. All the statistical analyses were performed using StatsDirect version 3.1.14. The sssays were performed in triplicate and the mean was used for statistical analyses. Statistical significance was determined using a one-way ANOVA test, followed by the post-hoc Tukey test. The Pearson correlation test was used to determine the significant correlations between oxidative stress biomarkers. The level of significance was set at  *p *< 0.05.

## Results

*The effect of ZnO on AChE activity*

As shown in Figure 2, AChE activity was significantly lower in CP group when compared with control group (*p *< 0.01). The groups which were treated with 0.1 and 1 μg/mL of ZnO NPs showed an apparent increase in AChE activity when compared with the CP group (*p *< 0.001). 10 and 100 μg/mL of ZnO NPs did not increase AChE activity that is probably because of toxic effects of ZnO NPs in these concentrations.

*The Effect of ZnO NPs on TAP*

As shown in Figure 3A, there is a significant decrease in control TAP by administration of CP (*p *< 0.05). In contrast, the groups which were treated with various concentrations of ZnO NPs increased the level of TAP. The most elevation was related to group II (1 μg/mL of ZnO NPs, 

*p *< 0.01).

*The effect of ZnO on TTM*

As seen in Figure 3B, the level of TTM in CP group in comparison with control group is decreased significantly (*p* < 0.001). There is an increase in all groups which received ZnO NPs, but in group II, TTM levels demonstrated a considerable increase in comparison to CP group (*p* < 0.01).

*The effect of ZnO on LPO*

There was a significant elevation in LPO (*p* < 0.001) in the CP group in comparison with control (Figure 3C). The groups which were treated with different concentrations of ZnO NPs showed apparent reduction in LPO when compared with CP group. The group, which was treated with 1 μg/mL of ZnO NPs caused the most reduction in LPO level in comparison with other groups.

*The effect of ZnO on MPO activity*

As depicted in Figure 3D, MPO activity increased in the CP group as compared to the control group (*p* < 0.05). Group II of ZnO NPs showed a significant decrease in MPO activity in comparison to CP group (*p* < 0.01), but there is no significant decrease in other groups.

*Correlations between oxidative stress biomarkers (TAP, TTM, LPO, and MPO* ***)***

The results of Pearson’s correlation analysis between oxidative stress biomarkers are presented in Figure 3E. The results of TTM were significantly positively correlated with TAP (*p* = 0.023), and negatively correlated with LPO and MPO (*p *= 0.002 and *p* = 0.04, respectively) results. In addition, although a negative correlation was observed between TAP and LPO levels, with a value of *p *= 0.007, there were no significantly correlations between these parameters and MPO activity.

*The effect of ZnO on TNF-α release*

As seen in Figure 4, TNF-α release significantly elevated in the CP group when compared to the control group (*p *< 0.05). A significant decrease in TNF-α levels was seen in the groups which were treated with 1 and 10 μg/mL of ZnO NPs as compared to CP group (*p *< 0.001 and *p *< 0.05, respectively).

*The effect of ZnO NPs on mitochondrial activity of lymphocytes*

To determine the mitochondrial activity of lymphocytes, after exposure to 75 μg/mL of CP and various concentrations of ZnO NPs (0.1, 1, 10, and 100 μg/mL), MTT test was done which are shown in Table 2. There is a significant decrease in viability of CP group (75 μg/mL) as compared with control group (*p < *0.01). The group, which was treated with 1 μg/mL of ZnO NPs with the combination of CP can significantly decrease the toxic effect of CP and increase the viability of cells in comparison with CP (*p* < 0.001) and control (*p* < 0.05) groups.

*The effect of ZnO NPs on caspase-3 and -9 activities of lymphocytes*

As it is shown in Table 2, CP significantly increased the activity of caspase-3 and -9 in comparison to control group (*p *< 0.001). The group, which received 1 μg/mL of ZnO NPs with the combination of CP could decrease the activities of caspase-3 and -9 when compared to CP group (*p *< 0.001 and *p *< 0.05, respectively).

*The effect of ZnO NPs on lymphocyte death (apoptosis vs. necrosis)*

As demonstrated in Figure 5, CP group showed a 30% decrease of live cells and 30% increase of necrotic cells in comparison to control group. Although all concentrations of ZnO NPs have been able to increase the percentage of viable cells, the most increase (33%) was seen at 1 μg/mL of ZnO NPs in comparison to CP group. Nevertheless, 100 μg/mL of ZnO NPs showed some more degrees of early apoptosis (Anexin V-FITC+/PI-, 12%), which is maybe due to toxicity of ZnO NPs in this concentration.

Human lymphocytes (1×10^6^/200 µL) were incubated with indicated concentrations of ZnO NPs and CP for 72 h and stained with Annexin V-FITC/PI as described previously. Quadrant analysis of fluorescence intensity of non-gated cells in FL1 (Annexin V-FITC) vs. FL2 (PI) channels was from 5,000 events. The values shown in the lower left, lower right, upper left and upper right quadrants of each panel represent the percentage of viable, early apoptotic, necrotic and late apoptotic (post-apoptotic necrotic) cells, respectively. The percentages of positive cells were indicated in each panel. 

## Discussion

In this study, CP which its oxidative stress, inflammatory and apoptotic effects have been proven in several studies (29, 30), was used for intoxication of human lymphocytes *in-vitro*. Then, the protective effects of ZnO NPs against CP-induced cytotoxicity, the effective dose of that, and recovery of toxic cells were evaluated by various tests. The obtained results indicate that ZnO NPs may have a beneficial role in the lowering CP toxicity by interfering with the biochemical and immunological pathways. 

It is well known that the major toxicity mechanism of CP is inhibition of AChE activity which caused production of ROS and oxidative stress (31). Co-treatment with lower concentrations of ZnO NPs (0.1 and 1 µg/mL) apparently increased AChE activity, but greater concentrations of that (10 and 100 µg/mL) seem to be toxic. Since inhibition of AChE by OP compounds is irreversible (32), any increase in the activity of this enzyme by NPs seems useful in neutralizing the negative effects of toxins. Hence, we can conclude that pharmacological dose of ZnO nanoparticles has a reversible potential to increase the AChE activity, an effect that is not seen in the toxic dose. In our previous studies, we demonstrated that metal oxide NPs such as CeO_2_ and MgO NPs in a special dose could increase cellular activity of AChE which is probably due to antioxidant potential of them (11, 26). There is a direct correlation between the activity of AChE and antioxidant capacity of cells and tissues, when one variable increases; the other also increases (33). Therefore, it can be argued that treatment of cells with ZnO NPs increases the activity of the AChE, which is due to an increase in the antioxidant capacity of cells. 

CP also can cause an increase in LPO level in human cells *in-vitro* (34), that is confirmed in this study. CP as a lipophilic substance may enhance LPO by direct interaction with cellular plasma membrane (35). Another mechanism is the induction of MPO activity as a heme protein that generates ROS and bioactive LPO products (36). In addition, according to the findings of Annexh-FITC and PI double staining, it was demonstrated that the treatment of lymphocytes with CP corresponded to approximately a 30% decrease in the number of live cells. In this context, previous studies on immune cells, including human Jurkat T cell line, monocyte like cell line (U937), and NK cells have shown that CP can induce apoptosis, and this apoptosis is mediated at least partially by the activation of intracellular caspase-3 (37-39), which is also depicted in this study by measuring caspase-3 and -9 activities. Besides, their findings explained that mitochondrial pathway is involved in CP-induced apoptosis, which is explored from the results of MTT assay. In addition, in another earlier study, it was indicated that CP at a concentration of 75 μg/mL exerts genotoxic effects in human lymphocytes, probably through DNA damage and chromosome breakage by the production of ROS (21, 40). In contrast, in this study it was found that low dose of ZnO NPs could counteract with the negative apopototic effects of CP. In the previous studies, it was demonstrated that high dose of ZnO NPs may cause cytotoxic effects through mitochondrial dysfunction, morphological modification, genotoxic and apoptotic responses in-vitro in different cell types such as human fetal lung fibroblasts (41), BEAS-2B and RAW264.7 cells (42), LoVo human colon carcinoma cell line (43), human liver cells (16), human skin melanoma cell line (A375) (18), rat retinal ganglion cells (44), and human alveolar adenocarcinoma cells (17). In this regard, Setyawati *et al*. represented that the elevated level of intracellular ROS at high concentration of ZnO nanomaterials (NMs) activated the apoptotic pathway through p53 while the induced ROS at low levels of ZnO NMs switched on the p53 dependent antioxidant mechanism to ensure better cell survivability and triggered the expression of antioxidant genes such as SOD2, GPX1, SESN1, SESN2, and ALDH4A1 to restore oxidative homeostasis (45). In support of this point, Zhang *et al*. indicated that ZnO NPs enhanced the tolerance to oxidative stress in mouse alveolar macrophages (MH-S) at a low concentration of 10 µg/mL or lower (46). In addition, Shoae-Hagh *et al*. demonstrated that ZnO NPs significantly ameliorate survival and function of rat pancreatic islets by reducing oxidative stress and preventing cells from undergoing apoptosis at lower concentration of 1/10 of LC_50 _(140 µg/mL) (47).

Collectively, although high doses of ZnO NPs (>10 µg/mL) are toxic to several types of cells, the results show that treatment with optimum doses of ZnO NPs (~1 µg/mL) can improve the viability and antioxidant capacity of cells. Therefore, ZnO NPs leads to the generation of ROS, which in high concentrations can cause oxidative stress and consequently cell death and in low concentrations it can stimulate the expression of antioxidant genes to restore oxidative homeostasis. In fact, observing such a diverse effect with both metals and nanomaterials is not surprising. For instance the toxicity of cadmium as a toxic heavy metal that induces oxidative stress (48), is dependent on the zinc homeostasis involving both zinc importers and zinc exporters. Therefore, cellular signal transduction pathways are influenced by zinc and redox status of the cell meaning the involvement of zinc transporters in cadmium cellular metabolism and induced oxidative stress (49).

Likewise, there are several studies suggesting that exposure of cells to CP increases the production of pro-inflammatory cytokines such as TNF-α that possibly mediated through JNK and p38 MAPK pathways (50, 51). In return, our findings showed that ZnO NPs inhibit inflammatory pathway by reducing the release of TNF-α from lymphocytes against CP. In line with these results, Ilves and his colleagues interestingly demonstrated that allergen-induced skin inflammation is significantly reduced by the exposure to ZnO NPs (52). They reported that treatment of atopic dermatitis mouse model with ZnO NPs suppresses local inflammation by down-regulation of the expression levels of pro-inflammatory cytokines such as IL-1β, IL-6, and TNF-α. It was also demonstrated that zinc as an antioxidant could reduce the levels of oxidative stress biomarkers and inflammatory cytokines such as TNF-α and IL-1β in patients with sickle cell disease (53, 54). Taken together, by adjusting/determining the optimum concentration of ZnO particles/Zn^2+^ ions, the positive anti-inflammatory properties of ZnO NPs can appear.

## Conclusion

This study show that treatment of lymphocytes with ZnO NPs induces anti-oxidative stress effect and decreases the mortality of the cells and level of factors involved in oxidative stress and inflammation. As a matter of fact, in lower concentrations of ZnO NPs, the change of toxic to non-toxic feature, recovery of mortality, and improvement of viability were confirmed. Therefore, in spite of induction of oxidative stress and toxicity by high concentrations (> 10 µg/mL) of ZnO NPs, these NPs exhibit positive antioxidant, anti-apoptotic, and anti-inflammatory effects at low concentrations (< 10 µg/mL) (Figure 6). Although, regarding different biokinetics of nanomaterials (54) occurrence of such effects is not unexpected, further studies are still needed to clarify the main mechanisms lying behind this protective effect of ZnO NPs.
